# Community-based organizations’ perspectives on improving health and social service integration

**DOI:** 10.1186/s12889-021-10449-w

**Published:** 2021-03-06

**Authors:** Etsemaye P. Agonafer, Savanna L. Carson, Vanessa Nunez, Kelli Poole, Clemens S. Hong, Maria Morales, Jessica Jara, Sarmen Hakopian, Tiffany Kenison, Ish Bhalla, Francesca Cameron, Stefanie D. Vassar, Arleen F. Brown

**Affiliations:** 1 Department of Health Systems Science, Kaiser Permanente Bernard J. Tyson School of Medicine, Pasadena, USA; 2 UCLA Division of General Internal Medicine and Health Services Research, Los Angeles, USA; 3 UCLA CTSI Community Engagement and Research Program (CERP), Los Angeles, USA; 4 David Geffen School of Medicine at UCLA, Los Angeles, USA; 5 Program in Medical Education Leadership and Advocacy (PRIME-LA), Los Angeles, USA; 6 Los Angeles County Department of Health Services (LAC DHS), Los Angeles, USA; 7 Olive View-UCLA Medical Center, Slymar, USA

**Keywords:** Medicaid populations, High-risk populations, Health and social service integration, Social determinants of health

## Abstract

**Background:**

Collaborations between health systems and community-based organizations (CBOs) are increasingly common mechanisms to address the unmet health-related social needs of high-risk populations. However, there is limited evidence on how to develop, manage, and sustain these partnerships, and implementation rarely incorporates perspectives of community social service organizations. To address these gaps, we elicited CBOs’ perspectives on service delivery for clients, the impact of the Whole Person Care-Los Angeles (WPC-LA) initiative to integrate health and social care, and their suggestions for improving health system partnerships.

**Methods:**

Using stakeholder engaged principles and a qualitative Rapid Assessment Process, we conducted brief surveys and in-depth semi-structured interviews with 65 key informants from 36 CBOs working with WPC-LA.

**Results:**

Major themes identified by CBOs included: 1) the importance of a holistic, client-centered, continuously engaged approach that is reliant on regional partnerships; 2) benefits of WPC-LA expanding capacity and networks; 3) concerns about communication and redundancy hindering WPC-LA; and 4) a need for more equitable partnerships incorporating their approaches.

**Conclusions:**

CBOs value opportunities for integration with health systems, bring critical expertise to these partnerships, and seek to strengthen cross-sector collaborations. Early, equitable, and inclusive participation in the development and implementation of these partnerships may enhance their effectiveness, but requires policy that prioritizes and incentivizes sustainable and mutually beneficial partnerships.

**Supplementary Information:**

The online version contains supplementary material available at 10.1186/s12889-021-10449-w.

## Highlights

- Community-based organizations (CBOs) highlighted the importance of a holistic, client-centered, continuously engaged approach that is reliant on regional partnerships;
- Although the CBOs described benefits of the integrative Whole Person Care-Los Angeles (WPC-LA) initiative, including expanded capacity and networks, they also expressed concerns that communication barriers and redundancies within the system hindered its aims; and
- CBOs endorsed a need for more equitable partnerships with health systems that incorporate their more holistic approaches

Ultimately, for health and social care integration efforts to address the needs of high-risk populations there must be policy that prioritizes and incentivizes sustainable and mutually beneficial partnerships.

## Background

Growing evidence demonstrates that adverse social factors contribute to poor health outcomes, high use of acute care services, and increased costs of care especially among high-risk populations that require complex care [1–3]. Healthcare systems, facing rising costs and payor incentives to improve population health outcomes and prioritize value, have made substantial investments in innovative programs that identify and address health-related social needs, particularly for their most vulnerable patients [4–6]. To screen patients for social needs and link them to appropriate resources, health systems often partner with community-based organizations (CBOs). These partnerships provide unique opportunities to address population health disparities while integrating care for individuals with medical and social needs [7].

Though increasingly common, health and social service collaborations that address physical, behavioral, public health and social needs, have limited evidence to guide their design, implementation, and sustainment [8–12]. Further, many existing partnerships are developed, funded, deployed, and evaluated by the healthcare sector, with little input from community stakeholders [7]. The lack of meaningful CBOs engagement may represent a missed opportunity, as these organizations are often embedded in the community and have an in-depth sociocultural understanding of the individuals and populations they serve.

Whole Person Care-Los Angeles (WPC-LA) is a 5-year, ~ $1.26 billion California Section 1115 Medicaid Waiver implemented by the Los Angeles County Department of Health Services (LAC- DHS) to provide and coordinate services for vulnerable Medi-Cal recipients. The 25 WPC-LA programs work to address the unmet health and social needs of six high-risk populations, including those experiencing homelessness, justice involvement, serious mental illness, substance use disorder, complex medical conditions, or barriers to a healthy pregnancy. The program initiated cross-sector CBO partnerships across LAC, with the goal of connecting individuals, identified in either community or healthcare settings, to services across the physical health, behavioral health, and social service continuum [13, 14].

This study used stakeholder engagement principles to elicit perspectives from CBOs regarding their approaches to providing services, the implementation of WPC-LA, and recommendations for improving the integration of health and social services.

## Methods

### Context

Our academic research team partnered with the WPC-LA leadership team. Study stakeholders were involved with all aspects of this research project: developing aims, designing pre-interview survey and interview guide, recruiting participants, validating and disseminating results. (For detailed methods, see Additional file 1: Appendix 1).

### Study sample

We identified CBOs that collaborated with WPC-LA through direct contracts, subcontracts, or informal non-contractual referrals to provide services such as housing, income benefits, vocational training, and substance use treatment. We recruited CBOs using snowball, purposive, and diversity sampling to achieve representation across the eight distinct LAC regions and six high-risk populations served by WPC-LA [15, 16]. Within these agencies, we interviewed key informants, defined as individuals with expert knowledge of the agency and its role in WPC-LA [17].

### Pre-Interview Survey & Interview Guide Development

We developed a pre-interview survey and individual semi-structured interview guide (see Additional file 1: Appendix 2 and 3). The pre-interview survey solicited CBOs’ characteristics and participants’ demographic characteristics. The semi-structured interview guide included open-ended questions exploring perspectives on clients served, agency’s approach to providing services to clients, and WPC-LA including facilitators and barriers to its successful implementation, and suggestions for improvement [18, 19].

### Data collection

Interviews were conducted in March 2019 to December 2019 and transcribed. Each transcript was checked for accuracy and de-identified. Data collection was complete when the team determined the sample was representative of CBOs across all regions of LAC and when thematic saturation was achieved in analysis [18].

### Pre- interview survey analysis

Survey data were entered into a REDCap database, and descriptive statistics were calculated.

### Semi-structured interview analysis

The interview transcripts were analyzed using the Rapid Assessment Process (RAP), a team-based qualitative inquiry for rapid turn-around of actionable results that informs infrastructure building and policy [20–23]. RAP utilizes a matrix of transcript summaries to identify major emergent themes and representative quotes across informants [20–24].

### Stakeholder engagement to validate results

We presented preliminary thematic results at an in-person conference and two virtual webinars with stakeholders (55 individuals, representing 20 CBO agencies). These meetings were held to verify the accuracy of, and obtain additional context for, preliminary themes and refine actionable recommendations.

## Results

We interviewed 65 participants representing employees from 36 CBOs that serve clients across every region of LAC (see Tables 1 and 2).

**Table 1:** Characteristics of Community Based Organization (*n* = 36)

	n (%)
**Organization Size**	
Small (1–10 staff members)	6 (16.7)
Medium (11–80 staff members)	13 (36.1)
Large (80–100+ staff members)	17 (47.2)
**Target Population**	
Mental Health	29 (80.1)
Homeless	28 (77.8)
Substance Use	26 (72.2)
Justice Involved	21 (58.3)
Medically Complex	18 (50.0)
Perinatal	15 (41.7)
Other (Legal assistance, youth service)	5 (13.9)
**Collaboration with other Agencies**	
To enroll clients in programs:	
Always/Very often	25 (69.4)
Sometimes	6 (16.7)
Seldom/Never	5 (13.9)
To deliver services to clients	
Always/Very often	32 (88.9)
Sometimes	3 (8.3)
Seldom/Never	1 (2.8)
**Funding Source**	
Los Angeles County Grants	21 (58.3)
Donations	11 (30.6)
Federal Grants	10 (27.8)
Private Grants	10 (27.8)
California State Grants	9 (25.0)
Other: Insurance Reimbursement for Services	4 (11.1)
No response	9 (25.0)
**Capacity Needs**	
***No needs***	4 (11.1)
***Internal Infrastructure Development***	
Staff recruitment, onboarding, and retention	18 (50.0)
Strategic planning for fundraising, policies and management	18 (50.0)
Quality Improvement	10 (27.8)
Facility upgrades or office expansion	10 (27.8)
Supporting diversity and equity in the organization	7 (19.4)
Recruitment and retention of board members	4 (11.1)
***Staff Development***	
Training to work with unique populations	14 (38.9)
Staff professional and leadership development/ Executive leadership coaching	14 (38.9)
Employee housing and social service needs	10 (27.8)
***Data Development***	
Information Technology infrastructure/ Improve technology and data privacy infrastructure	13 (36.1)
Knowledge on data sharing and compliance	11 (30.6)
Evaluation of Service Delivery	7 (19.4)
***External Infrastructure Development***	
Support and technical assistance for county contracts	11 (30.6)
Coalition and collaboration development	7 (19.4)
Support for subcontracting organizations providing subcontracts	6 (16.7)

**Description:** Characteristics of CBOs including its size, target population, collaboration with other agencies, funding sources and capacity needs

**Footnotes:** Authors’ analysis of data from pre-interview survey administered to participants. Responses from individual participants were aggregated. CBOs could report serving more than one target population, funding source and capacity need

**Table 2:** Characteristics of Participants (*n* = 65)

	n (%)
**Role in Community Based Organization**	
Frontline provider	24 (36.9)
Management	25 (38.5)
Executive	16 (24.6)
**Female**	48 (73.9)
**Race/Ethnicity**	
White	23 (35.4)
Hispanic/Latino/a/x	22 (33.9)
Black/African American	15 (23.1)
Asian/Pacific Islander	6 (9.2)
Native American/American Indian	1 (1.5)
**Highest level of education**	
High School Graduate	4 (6.2)
Some College	10 (15.4)
College (BS/BA)	13 (20.0)
Masters/Graduate	31 (47.7)
Professional School (JD)	5 (7.7)
Missing	2
**Experience:**	
Mean years in social services field (*n* = 61)	12.4 (SD 9.5)
Number of years working with the agency	
< 1 year	8 (12.3)
1–5 years	28 (43.1)
> 5 years	29 (44.6)
Number of individuals who live in region agency serves	29 (44.6)
Number of employees who shared lived experiences with their clients	23 (35.4)

**Description**: Characteristics of participants including their role in their CBO, sex, race/ethnicity, education level, and experience

**Footnotes:** Authors’ analysis of data from pre-interview survey administered to participants

### Agency demographics

All agencies reported serving multiple WPC-LA target populations. A majority reported they “very often” or “always” collaborated with other agencies to enroll clients in programs (69.4%) and to deliver services to clients (88.9%). The majority (88.9%) identified multiple capacity needs, including internal infrastructure, staff, and data development.

### Participant demographics

On average, there were 2 participants per agency (range: 1–6). Participants represented frontline providers who worked directly with clients (e.g., community health workers (CHWs)), managers responsible for directing specific programs and employees, and agency executives (e.g., chief executive officers). Participants reported a mean of 12.4 years (SD 9.5) experience working in social services, and 44.6% reported working at their agency for over 5-years, while 44.6% lived in the community served by the agency. Many participants (35.4%) reported self-defined “shared lived experiences” with clients; for example, they had a personal or family history of experiencing homelessness, substance use, and/or justice involvement.

### Interview themes

Participants described common strategies for service delivery, including client engagement, needs assessment, referral to and fulfillment of comprehensive care. Additionally, participants described perspectives regarding the strengths and challenges with the WPC-LA partnership and shared recommendations for strengthening health and social service integration. (see Table 3).

**Table 3:** Major Themes and Quotes

**CBO Service Delivery Process**	
**Client-centered-** Holistic understanding of clients and communities	*“...the [clients’] lack of community often creates a level of fear, anxiety, and distrust that makes us have to engage this individual at least a half-dozen times before we can have a simple breakthrough like eye contact or, for that matter, dialogue.”* – Frontline provider
**Continuous system-** multiple entry points, active outreach and engagement efforts, and comprehensive needs assessment based on clients’ priorities and eligibility for programs	*“Because of the vulnerability of the people that we work with …. establishment of eye contact, and conversation is necessary for anything to begin. … in order to establish a level of contact, which is very seriously missing … .it’s important to understand that engagement with individuals relies on a consistent approach that does not provide barriers or judgement or rules that more often than not people have encountered when approaching service providers. … Start from a basic, very human level of understanding where people can communicate with each other pretty easily, we are confident those engagements will open a series of doors that will allow for us to better understand what a person needs.”* – Executive “… *it’s about going to them and being consistent and building the trusting relationship”* – Management “… *connecting with them where they’re at. Providing them with whatever service they need in the moment, and sometimes it’s just a cup of coffee and a bag of beef jerky*.”- Frontline provider *“...walk them down the path, get them document ready, get them socially ready, get them ready mental health-wise …. Get them clean and sober.”* – Frontline provider
**Dependent on regional partnerships-** service network to meet comprehensive clients’ needs	*“If we refer somebody … we are there to support them and* vice versa *… agencies are really collaborating … there’s a system”* – Management *“We’re built on collaboration, so we’re built as an organization to be a collaborative group. So, we divide up the cities in our area and say different organizations are the lead … [We] have multiple avenues of case conferencing, different committee work … we conference on our outreach clients … talking about who’s had interaction with these individuals? What’s the progress that you’ve seen? What are the challenges that you’ve seen? Oh, we’ve been able to make movement here. Okay, so it sounds like you have a better connection with that client; how do we support you in that instead of trying to be a different connection?*” – Management *“sometimes … we might be providing the same services [as another agency], but there might not be connection between the worker and the clients. So whoever this person feels comfortable working with … (s) he has a chance to kind of choose. Because before it was like you work with one agency and you didn’t like the person, and sometimes that was the challenge because they didn’t want those services because there was no connection.”* – Management
**Impact of WPC-LA Partnerships**	
**Benefits:** • Expand partnership networks • Funding for social care programming (new services/resources) • Employing those with lived experiences (Collaboration Team, patient navigators, and CHWs)	*“Conceptually, the idea behind Whole Person Care, being able to … Be the person to kind of hand hold them through connecting with some of these resources. We thought that was brilliant. Brilliant!”* – Frontline provider *“[WPC-LA programs] are taking the time to address all of these various groups of individuals that are a very vulnerable population. …. to try and create a system of care to address those needs …. that is fantastic and really necessary.”* – Executive *“[WPC_LA is] one of the best changes that we’ve had in the justice system. Somebody finally woke up and said, “Look, what’s happening isn’t working. The way with things are, isn’t working.” I’m really, really happy to be a part of this community and to help it grow … I think it’s a new approach that is long overdue. It’s long overdue.”* – Management *“the community health workers, who they are employing …. When I mention that to prospective clients … [that they will be] working with somebody who has lived experience and is just paying it forward and now they’re employed and—you could see their reaction … To feel like you can relate and somebody that can relate to you—you can’t measure that.”* – Management
**Challenges:** • Communication issues • Overlap with CBOs work • Continued limitation of “end” resources	*“it was a little confusing in the beginning. It was a new program and so it wasn’t, like, clearly defined what exactly they were doing”* – Management *“the lack of quality referrals …. we’re targeting the most vulnerable folks and it’s like these folks are already in bad spaces. It doesn’t take a whole lot to disappoint a disappointed person. You know, there’s a description in the Bible that says, “Hope deferred makes the heart sick, but a desire accomplishes a tree of life.” And I think we’ve deferred a lot of hopes.”* – Executive *“[they are] still trying to figure out what they can support us in that’s different than what we already do.”* – Management *“We often don’t feel that our voice is heard and that we are often dealing with the consequences of decisions that are made by others that force us to have to restructure or retool what we do.”* – Executive *“if the resources were actually out there, then we’d probably go straight to that resources as opposed to using them [WPC-LA} as a middleman.”* – Management *“there’s all this emphasis on, quote, “systems” and “structures,” but none of this works unless I know somebody else’s name. … always dissolving into personal relationships, but also because of contracts and because of the official rhetoric around things, having to … give the illusion of a functioning system … .this isn’t a “system”, especially if getting somebody connected to services … is different every single time. You cannot predict timelines … predict quality of service …. hold anybody accountable …. really when people say “system,” I think what they actually mean is … very slowly prioritized services … prioritization are really just conversations about lack of resources …. you wouldn’t need to prioritize people for anything if it was all available …. conversations about systems are conversations about priority, which are ultimately conversations that you’re having when you’re not having a conversation about the allocation of money and resources.”-* Management
**Suggestions for Partnership Improvement**	
• Build a more equitable partnerships with the healthcare sector • Improve communication • Advocate for continued funding to support their integrative work	*“we need other parts of the system to radically change and alter what they do … unlearn what they’ve done for 50 years and retool themselves to a new approach.”* – Executive *“It’s hard for anybody to embrace change and so if you have a consistent figure, someone that you trust and that … who values the very things that we talked about as effective, collaboration, training, empowerment, consistency is really helpful. If WPC-LA wants to be successful, they need to really invent themselves in communities, show up to meetings, participate and be consistent.”* – Management *“I would implore [WPC-LA] to … understand that the dedication of the people who do this work … we are in need of dialogue, inclusion, and some seat at the table in order to share … [the] perspective from the frontlines.”* – Executive *… the “more people you have working in the system that do not have a relationship with the people on the street, the worse that system is going to function with people on the street.” –* Frontline provider *“Let’s bring the resources … bring the Whole Person Care team actively into the community but let’s also make sure that the professionals in each area knows who their Whole Person Care person is, has access to them and that they also know because services are changing all the time … so they know what’s going on”* – Management *“my biggest thing would be WPC-LA possibly asking the county for … more resources … people in the community are in need of stuff that we can directly hand to them.”* – Management

**Description:** Major themes and quotes identified from semi-structured interviews including CBO service delivery process, impact of WPC-LA partnership, and suggestions for partnership improvement

**Footnotes:** Authors’ analysis of data from semi-structured interviews with participants

#### CBOs’ service delivery process

The CBOs described a multi-pronged approach for engaging clients in social services. CBOs commonly portrayed a holistic, client-centered, and continuous service delivery process. They employ continuous engagement to build relationships with clients, identify their needs, and provide wrap-around services that relies on their regional network.

##### Holistic understanding of clients & continuous engagement

Participants described their clients as individuals with diverse cultural and social influences who had multiple overlapping clinical and social needs—fundamentally; they expected their clients to be “*complex*.” As a frontline provider noted, “*99.9% of my clients are homeless, on the streets, severely mentally ill, not taking medication, not receiving mental health services, and substance abusers.*” While some participants struggled with listing clients’ strengths, they consistently noted an appreciation of their clients’ resiliency, adaptability, and resourcefulness in the face of various availability to resources by neighborhood. As one executive described, *“[clients have] the ability to take nothing... and be determined long enough to stay the course for that nothing to become something, even if it’s just surviving in very hostile environments*.” Participants described the variability of resources by neighborhood, noting, *“clients...just live in the wrong zip code, they kind of fall through the cracks.*”

This comprehensive understanding of clients and their environments informed the manner in which they delivered services to their clients. Participants consistently described this process as having multiple entry points (e.g., walk-in, referral, street outreach) that included continuous engagement methods like building trusting relationships with clients and providing entry services (e.g., group counseling or classes). As one executive described, building trust to understand clients’ priorities involves, “*establishment of eye contact; … conversation is necessary for anything to begin; … a consistent approach that does not provide barriers or judgment; … a basic, very human level understanding*.” Participants reported that once they establish rapport, they address client needs through formal and informal assessments, program eligibility criteria, and, most importantly, clients’ priorities. As one frontline provider described, *“as … goals are met, then we start to determine other goals … to help them find whatever they need.”*

##### Reliance on regional partnerships for wrap-around services

Since CBOs typically have a specific focus, once trust is built with a client, participants described relying on regional partnership networks with other agencies to address clients’ needs. An agency focused on addressing homelessness may partner with other agencies to deliver additional services like legal assistance or income benefits. Agencies described using their local expertise and known networks of available social services to provide warm-handoffs and referrals that would work best for clients. As one manager described, “*sometimes … we might be providing the same services [as another agency], but there might not be a connection with the client. So, whoever [the client] feels comfortable working with … [they can]choose.”* Participants described collaboration with other agencies as a critical, continuous process of relationship-building that developed over time at regional meetings, case conferences, and community events. As another manager stated, “*We don’t tend to think that we can solve a client’s problem... internally and solely.”*

#### CBO’s perspectives on the WPC-LA partnership

##### Benefits of partnerships

Among the benefits described by participants was the emphasis on WPC-LA expanding regional partnership networks, funding social care programming, and employing those with lived-experience. Many participants described how partnering with health systems was new and evolving, however, emphasized that cross-sector partnership would enable them to comprehensively serve their clients’ diverse needs.

##### Diverse network of WPC-LA programs that expanded services

Participants expressed enthusiasm for the goals of WPC-LA. The participants viewed the number and diversity of WPC-LA programs as both an important recognition of and response to their clients’ complex, wide-ranging health and social needs. They also valued new opportunities to expand their regional partnership networks through work with multiple county departments, healthcare systems, and other CBOs. One executive expressed strong support for the program’s willingness to take “*the time to address a very vulnerable population … create a system of care … that is fantastic and necessary*”. A manager similarly described, *“somebody finally woke up and said, ‘Look, the way things are isn’t working’ … it’s a new approach that is long overdue.”*

##### Expansion of CBO capacity

Several participants reported that WPC-LA funding expanded their service capacity by supporting new programming and enhancing existing program with increased staff. Homeless agencies, for example, indicated that WPC-LA funds expanded their ability to staff the multidisciplinary “street outreach teams,” deployed to homeless encampments across the county to provide health, mental health, case management, and social work services. Other participants described co-located benefits programs that screened and enrolled eligible clients for income or disability benefit assistance. Moreover, several CBOs received support to hire CHWs either directly through LAC DHS or indirectly via contracts. Participants frequently singled out these CHWs as valuable because of their relatability and commitment to individuals and community that enabled CBOs to engage with even the hardest to reach clients. As one frontline legal services provider described, *“while defending [my clients’] eviction, their CHWs were able to locate housing … came with us to court … helped [them] understand the terms of their settlement … and... move [them] to their new place without an eviction on their record.”* An executive noted that CHWs, *“changed the number of individuals that come through …*. *because when they talk to [clients], they are sincere about what they’re trying to do.”*

##### Challenges to partnership

Various challenges to partnerships within WPC-LA were described including a lack of clarity about programming, concern for overlapping processes, and a need to increase available resources and services. These challenges contributed to the CBOs’ wariness of WPC-LA’s ability to fully support clients.

##### Lack of understanding of WPC-LA programs and implementation protocols

Participants described a general lack of knowledge about available programs, including services offered and eligibility. They also reported an inability to communicate directly with WPC-LA staff about clients due to an impersonal referral process. Interviewees attributed some of the confusion to the presence of multiple WPC-LA programs at different stages of implementation for overlapping high-risk groups, but much of the concern focused on an impersonal, unilateral referral process conducted mainly through phone messages. Generally, participants described a lack of personal contact to discuss client cases, which made it difficult to understand program eligibility criteria, client pathways, and progress of referred clients. Several participants reported that electronic handoffs lacked important client data (e.g., accurate contact information, needs assessments) and did not include a contact to clarify client circumstances. A frontline provider explains, “*there’s still not ‘the seamless integrated process’—we get referrals, and we have no information; we don’t know where to pick up, so how do we … coordinate and integrate a plan and a client for success?*”

##### Potentially overlapping processes

The uncertainty about the WPC-LA referral process was exacerbated by ambiguity about which direct services were available. Participants expressed concern about whether WPC-LA was another “middleman” referral service, duplicating work currently done by the CBOs, or if it provided new direct services. As one manager stated, we are “*still trying to figure out what [WPC-LA] can do to support us … that’s different than what we already do. Because a lot of my staff … say ‘Well, I can do it faster, so I’m just going to do it.’*” This concern was most prominent among participants at CBOs serving homeless populations, who noted that wait times for housing were dependent on one county-wide housing application process.

##### Limited “end” resources

Some participants expressed reluctance to entrust their clients to WPC-LA, as it was unclear if the program provided any additional “end” resources, such as housing or substance use treatment. For example, enrolling individuals with substance use disorder into WPC-LA programming did not always expedite their placement into inpatient treatment because most facilities were at capacity, and availability differed across county regions. A frontline provider explains, *“[clients] count on us … we tell the clients we are here to help you … and once we do link them and they don’t get the appropriate help … they’re discouraged because... ‘it’s another fake promise.’”*

#### Suggestions to improve WPC-LA partnership

Despite the challenges participants identified, many were eager to build more equitable partnerships with the health sector, improve communication across sectors, and advocate for continued funding to support integrative medical and social care.

##### Build equitable partnership

Participants strongly argued for a true partnership in WPC-LA to achieve consistent and equitable bi-directional knowledge exchange that improves care and outcomes for shared clients. Participants described not being included in the design and implementation of the program. As a result, their expertise and experience—in local neighborhood assets and needs, service networks, population-specific knowledge, cultural awareness, and active engagement strategies—was not incorporated. As an executive advised, *“[CBOs need] dialogue … inclusion … and a seat at the table …*. W*e often don’t feel that our voice is heard, and … we are often dealing with the consequences of decisions that are made by others that force us to have to restructure or retool what we do*.” Another executive suggested both sectors need to “*unlearn what they’ve done for 50 years and retool themselves to a new approach,*” one that is more client-centered and community-driven.

##### Improve communication

CBOs recommended improving communication through more collaborative client engagement, a streamlined electronic referral processes that shares accurate client information without redundancy, and regional meetings with partnered organizations to determine eligibility and enrollment. As a manager described, *“if WPC-LA wants to be successful, they need to really invest themselves in communities, show up to meetings, participate and be consistent.”*

##### Advocate for funding

A majority acknowledged the need for increased funding to build their organizational capacity to continue and expand this work. The participants also highlighted variation in the resources allocated to different cities in their regions and requested additional end resources to areas with shortages, such as job opportunities, housing, or inpatient substance use treatment.

Based on these CBO suggestions, we outline policy recommendations for health and social service integration (see Table 4).

**Table 4:** Policy Recommendations for Health and Social Service Integration

• Incentivize and prioritize a step-wise approach to cross-sector, mutually-beneficial, community-driven partnerships that use broad longitudinal stakeholder engagement to establish agreements for shared governance, accountability, funding, data, program implementation. This step-wise approach to achieving non-hierarchical partnership will require cross-sector training, but includes o Step 1: Identify and understand individual partners’ values, priorities, goals, funding streams and care delivery processes o Step 2: Align and streamline processes to create shared accountability and mutually beneficial return on investments o Step 3: Co-create and implement integrative programming design and evaluation with an emphasis on incorporating partners priorities, including measures demonstrating whether or not there is a reduction in duplicative work and cross-sector cost savings o Step 4: Co-evaluate the initiative with the aim to improve the partnership and enhance bidirectional learning and knowledge • Enhance existing cross-sector partnerships by o Mandating use of stakeholder engagement principles to communicate and outline non-duplicative and integrative programmatic goals, plans, implementation, and evaluation process o Building capacity through investments in CBOs organizational capacity, establishment of a universal consent form, and building an information technology platform that can be used across sectors and data reporting system • Increase funding for programs that integrate medical care and social care and align with other relevant sectors (e.g. agriculture, transportation, etc.) to provide more comprehensive funding for the resources need for specific programming (i.e. housing, benefits, access to health/mental health care)	

**Description**: CBOs’ perspective on policy recommendations for improved health and social service integration

**Footnotes: **Authors’ analysis of data from semi-structured interviews with participants

## Discussion

The CBOs’ approach to engaging clients, their views on WPC-LA, and their suggestions for improving the program add essential dimensions to research on health and social service partnerships that provide care for medically and socially complex populations. CBOs, instead of categorizing their clients into high-risk populations, take a holistic approach to client care that includes their community context. They view their service delivery as relational rather than transactional and participate in long-standing networks with other agencies. While there was enthusiasm for the WPC-LA collaboration, it was tempered by concerns about their expertise not being included in the program’s design and implementation. CBOs suggested several strategies to incorporate their expertise by building and maintaining collaboration through equilateral power-sharing and two-way capacity building.

Our findings are consistent with the limited literature suggesting CBOs are optimistic about partnering with healthcare systems to better serve the clinical and social needs of vulnerable populations [25]. Participants placed value on WPC-LA’s ability to expand networks and capacity for clients, agencies, and health system collaborators. However, consistent with existing frameworks, they also described financial and organizational barriers to effective implementation and sustainment of healthcare-social care partnerships [26, 27], among them different approaches to eliciting, categorizing, and addressing client needs, competing priorities and goals, and financial and operational barriers to comprehensive, efficient, and effective partnerships. These challenges reinforce fundamental critiques of healthcare-led integration that are not yet incentivized to breakdown the cross-sector silos to create population-level solutions that address sensitive, individual-level issues such as housing instability, hunger or trauma. To achieve better alignment between health and social care, there is a need for intentional inclusion of CBOs’ ability to understand an individual’s plight in the context of their community into the design and implementation of these programs.

Policies are needed that incentivize the process of building early and consistent cross-sector partnerships that places value in building trust, reducing redundancies, addressing gaps in services, and improving communication across multiple dimensions, including client-centered service delivery, finances, data sharing, and metric reporting. Policies must also address features of governmental funding opportunities that can limit participation of the diverse and potentially complementary voices of CBOs, among them rapid turnaround times and inadequate attention to client complexity and interconnected needs. The request for “*dialogue … inclusion... and a seat at the table*” reflects the need to accelerate progress on the spectrum of partnership between sectors, from coordination to collaboration to full integration [28–32]. In order to achieve the step-wise approach described in Table 4, there is a need for inclusive, interprofessional training on how to operationalize strategies and best practices of non-hierarchal partnership.

Although WPC-LA is the largest California Section 1115 Medicaid waiver in the second-largest safety net system in the country, this study focuses on the perspective of one group of stakeholders in a complex program with many active participants. We attempted to mitigate this limitation by sampling across all regions of LAC, and high-risk populations served to be reflective of CBOs across the nation and validating preliminary themes with CBO representatives who were not interviewed for the study. Additional research should be done to identify client-centered, community-driven strategies and best practices to not only shift from health care system priorities, but to educate cross-sector professionals on how to participate in equitable partnerships.

## Conclusion

CBOs expertise in serving marginalized populations can be central to enhancing the effectiveness of health and social service efforts, but their expertise is not routinely included in the design and implementation of integrative programs. This study suggests that expanding these integrative models of care requires targeted and inclusive training, funding, shared planning, governance, and intentional program implementation to prevent unintended consequences of a siloed, single-sector approach. To create an effective infrastructure that tackles complex public health issues, it is critical to create comprehensive policies that simultaneously build relationships, incorporate the assets of communities, and address the diverse needs of all stakeholders. It is through these upstream policy changes that downstream lessons can be learned to shift the paradigm of care into the true partnered approach sought by communities in need.

## Supplementary Information

**Additional file 1: Appendix 1**. Detailed Methods. **Appendix 2**. Pre-Interview Survey. **Appendix 3**. Semi-Structured Interview Guide.

## Data Availability

All data generated or analyzed during this study are included in this published article and its supplementary information files. For any requests about the data from this study, please contact Etsemaye P. Agonafer at Etsemaye.P.Agonafer@kp.org.

## Abbreviations

CBO: Community-Based Organizations
WPC-LA: Whole Person Care-Los Angeles
LAC: Los Angeles County
DHS: Department of Health Services

## References

[1] Braveman P, Gottlieb L (2014). The social determinants of health: it's time to consider the causes of the causes. Public Health Rep.

[2] Palmer RC (2019). Social determinants of health: future directions for health disparities research. Am J Public Health.

[3] Woolf SH, Braveman P (2011). Where health disparities begin: the role of social and economic determinants--and why current policies may make matters worse. Health Aff (Millwood).

[4] Horwitz LI (2020). Quantifying health Systems’ Investment in social determinants of health, by sector, 2017-19. Health Aff (Millwood).

[5] Muhlestein D , S.R., Richards R , McClellan MB., Recent progress in the value journey: growth of ACOs and value-based payment models in 2018. , in Health Affairs Blog [on the Internet].

[6] Bachrach D, Wallis K, Lipson M, P.H (2014). Manatt Health Solutions., Addressing patients’ social needs: an emerging business case for provider investment.

[7] Fichtenberg C (2020). Health and human services integration: generating sustained health and equity improvements. Health Aff (Millwood).

[8] Siegel B (2018). Multisector partnerships need further development to fulfill aspirations for transforming regional health and well-being. Health Aff (Millwood).

[9] Woulfe J (2010). Multisector partnerships in population health improvement. Prev Chronic Dis.

[10] Zahner SJ, Oliver TR, Siemering KQ (2014). The mobilizing action toward community health partnership study: multisector partnerships in US counties with improving health metrics. Prev Chronic Dis.

[11] Institute, K.G (2018). Investing in social services as a Core strategy for healthcare organizations: developing the business case.

[12] Griffin K, N.C, Realmuto L, Weiss L. Partnerships Between New York City Health Care Institutions and Community-Based Organizations. New York: Grerater New York Hospital Assocation The New Yor Academy of Medicine; 2018.

[13] (DHS), L.A.C.D.o.H.S. *Whole Person Care-Los Angeles (WPC-LA)*. [cited 2018 August 15 ]; Available from: http://dhs.lacounty.gov/wps/portal/dhs/wpc/.

[14] Services, C.D.o.H.C. Whole Person Care Pilots. [cited 2018 August 15]; Available from: http://www.dhcs.ca.gov/services/Pages/WholePersonCarePilots.aspx.

[15] Palinkas LA (2015). Purposeful sampling for qualitative data collection and analysis in mixed method implementation research. Admin Pol Ment Health.

[16] Goodman LA (1961). Snowball sampling. Ann Math Statist.

[17] Marshall M (1996). The key informant technique. Fam Pract.

[18] Rubin HJ, Rubin I (2012). Qualitative interviewing : the art of hearing data.

[19] Britten N (1995). Qualitative interviews in medical research. BMJ.

[20] Hamilton A. Qualitative methods in rapid turn-around health services research: VA HSR&D National Cyberseminar Series. Los Angeles: Spotlight on Women’s Health; 2013.

[21] Hamilton AB, Finley EP (2019). Qualitative methods in implementation research: an introduction. Psychiatry Res.

[22] Kirk MA (2016). A systematic review of the use of the consolidated framework for implementation research. Implement Sci.

[23] Averill JB (2002). Matrix analysis as a complementary analytic strategy in qualitative inquiry. Qual Health Res.

[24] Miles MB, Huberman AM, Saldana J (2014). Qualitative Data Analysis : a Methods Sourcebook.

[25] Byhoff E, Taylor LA (2019). Massachusetts Community-Based Organization Perspectives on Medicaid Redesign. Am J Preventive Med.

[26] McGinnis T, Crawford M, Somers SA (2014). A state policy framework for integrating health and social services. Issue Brief (Commonw Fund).

[27] Amarashingham R (2018). Using Community Partnerships to Integrate Health and Social Services for High-Need, High-Cost Patients. Issue Brief (Commonw Fund).

[28] Pahwa R, Smith ME, Kelly EL, Dougherty RJ, Thorning H, Brekke JS, Hamilton A. Definitions of Community for Individuals with Serious Mental Illnesses: Implications for Community Integration and Recovery. Adm Policy Ment Health. 2021;48(1):143–54. 10.1007/s10488-020-01055-w. PMID: 32504269. 10.1007/s10488-020-01055-w 32504269

[29] Dzau VJ (2017). Vital directions for health and health care: priorities from a National Academy of medicine initiative. JAMA.

[30] Trust, H.R.E. A Playbook for Fostering Hospital–Community Partnerships to Build a Culture of Health. Chicago: Robert Wood Johnson Foundation; American Hospital Association; 2017.

[31] Begins H (2019). Levels of health related social needs and social determinants of health integration framework.

[32] Burke GC, Shearer C, R.-C.K (2019). Complex Construction: A Framework for Building Clinical-Community Partnerships to Address Social Determinants of Health.

