# Supplementary material for: Community-based organizations’ perspectives on improving health and social service integration
Source: BMC Public Health. 2021 Mar 6;21:452. doi: 10.1186/s12889-021-10449-w (PMC7937223; doi:10.1186/s12889-021-10449-w)
Supplement: Supplementary file 1 — Additional file 1: Appendix 1. Detailed Methods. Appendix 2. Pre-Interview Survey. Appendix 3. Semi-Structured Interview Guide. [file 12889_2021_10449_MOESM1_ESM.zip › additional file(s)/Appendix 1. Detailed Methods_ESM.docx]

**Appendix 1. Detailed Methods**

*Context*

WPC-LA has a leadership team which includes a Clinical Team and a Collaboration Team. The Clinical Team conducts outreach to hospitals and affiliated clinics across Los Angeles County to engage patients in clinical care. The WPC-LA Collaboration Team facilitates regional relationships between the health systems participating in WPC-LA and community organizations that link patients with social resources and care across Los Angeles County. Our partnership included the academic research team, WPC-LA leadership (author CH) and members of their Collaboration Team (author KP; acknowledgements CH, MH, AJ, DL, MR, SS, DZ). We collaborated with the WPC-LA leadership to conduct a partnered evaluation of the program’s implementation. Study stakeholders were involved with all aspects of this research project from developing the aims, survey design and interview guide, recruitment of participants, validation of results and dissemination.

*Study Sample*

We identified CBOs that partnered with WPC-LA, through direct contracts, subcontracts, or informal non-contractual referrals. Given the diversity of CBOs in LAC, we recruited organizations using snowball, purposive, and diversity sampling to achieve representation of agencies across all eight distinct regions of the 4300 square miles of LAC called Service Planning Areas and all six high-risk populations served by WPC-LA.[15, 16] Within these agencies, we interviewed key stakeholders, defined as individuals with expert knowledge of the agency and its role in WPC.[17, 34] Recruitment involved an introductory email to a contact within the organizations describing the aims of the study, after which a follow-up telephone conversation was scheduled to answer questions, determine willingness to participate, and identify key stakeholders.[34] We did not limit the number of participants from an agency as we wanted a diversity of perspectives by agency position. Of the 80 organizations contacted, 48 (60%) agreed to have a telephone conversation with the study team, and 36 of those organizations (75%) agreed to participate in data collection. Key stakeholder participants (n= 65) included individuals who served as frontline providers (e.g., community health workers), managers (e.g., program coordinators), or executives (e.g., chief executive officers).

*Pre-Interview Survey & Interview Guide Development*

Together, the WPC-LA team and the academic researchers developed and fielded a pre-interview survey and prepared an individual semi-structured interview guide based on a literature review of collaborations between health and social service agencies. The pre-interview survey solicited characteristics of the agency (i.e., size, years active, funding source, organizational readiness to participate in WPC-LA, capacity-building needs), demographic characteristics of the participant (i.e., race, years of experience, lived experience). The semi-structured interview guide included open-ended questions that explored perspectives on 1) clients served, 2) agency vision, goals, and approach to providing services to clients, and 3) their views on the WPC-LA program, including facilitators and barriers to its successful implementation (See Appendix). [18, 19] We conducted pilot surveys and semi-structured interviews with WPC-LA program staff and retired employees at community social service agencies (n=3) to refine language and content before administration to participants. These surveys and interviews were not included in the analyses.

*Data Collection*

The academic research team conducted interviews from March 2019 to December 2019 until thematic saturation was reached in the data analysis phase.[18, 19] Interviews took place in-person at a location that was convenient to the participant, typically at the participant’s agency location. The interview and survey collection usually occurred in a private office. Interviews lasted between 30 to 60 minutes. Interviews were recorded and professionally transcribed. Each transcript was checked for accuracy and de-identified.

Five members of the academic research team received training in semi-structured interview techniques; the interviewers included three physicians, one medical student, and one research associate (authors E.A., V.N., M.M., I.B., T.K.). The training occurred in three phases: review of an audiotaped mock interview to standardize approaches, observation of an expert interview, and conducting an interview while observed by another team member. Data collection was complete when the larger stakeholder/academic team determined that the sample was representative of CBOs across the regions of Los Angeles County and when thematic saturation was achieved in the analysis. [18, 19]

The Los Angeles County Department of Public Health IRB approved the study.

*Pre- Interview Survey Analysis*

Survey data were entered into a REDCap database by two research assistants with double data entry and then analyzed using descriptive statistics.

*Semi-Structured Interview Analysis*

The interviews were systematically analyzed by five team members trained in Rapid Assessment Process (RAP) (authors E.A., V.N., T.K., I.B., SC). This method uses a team-based qualitative inquiry for rapid turn-around of actionable results that informs infrastructure building and policy.[20, 21] RAP is a component of the Sort and Sift, Think and Shift method where "diving in" and "stepping back" phases are interdependent and synergistic. [20, 22, 35]During the "diving in" phase, the transcripts were summarized, and powerful quotes were identified and inventoried using a structured template organized by key domains derived from the interview guide and the Consolidated Framework for Implementation Research (CFIR).[23]

To ensure consistency and reach consensus on the summarization process and inclusion criteria for each domain, two members of the analysis team summarized the first six interview transcriptions independently, after which three meetings were held to assess styles across the team and format of the summary template, and to reconcile any discrepancies in summaries.[20, 21, 24] After consistency was established, the remaining transcripts were divided across the team for summarizing. Data from the summaries were transferred to a matrix that further organized key constructs from the CFIR to identify emergent themes and representative quotes across groups of informants. [20, 21, 24]

We used four constructs of CFIR because it uses a taxonomy, terminology, and definitions that allow for a holistic exploration of implementation factors as they relate to our domains of interest, i.e., how CBOs view their clients, service delivery process, and impact of WPC-LA. The CFIR constructs used included: outer setting (e.g., patient needs and resources), inner setting (e.g., culture, networks, and communication), process (e.g., evaluation), and intervention (e.g., adaptability and complexity). During the "stepping back" phase, major themes are reviewed broadly to determine which domains lend themselves to more exploration to further aid in improvement efforts. Summary points were transferred into matrices[24], in order to facilitate comparison across participants, organizations, and CFIR constructs and domains. Matrices gave the team a form of data display which allowed them to explore and evolve potential themes.[25] During the "stepping back" phase, the matrix was used to review summary points broadly, identify major themes, and determine which domains lend themselves to more exploration.

*Stakeholder Engagement to Validate Results*

An in-person partnered conference and 2 electronic webinars were held with participants and WPC-LA leadership to 1) verify accuracy of the preliminary themes and summary recommendations, 2) obtain additional context for preliminary themes identified, and 3) gain insight to refine actionable recommendations that can inform the integration of medical and social services in the WPC-LA program. Collectively 55 individuals representing 20 agencies participated in the in-person or webinar presentations of preliminary results to clarify and verify the major themes.
