# Supplementary material for: Community-based organizations’ perspectives on improving health and social service integration
Source: BMC Public Health. 2021 Mar 6;21:452. doi: 10.1186/s12889-021-10449-w (PMC7937223; doi:10.1186/s12889-021-10449-w)
Supplement: Supplementary file 1 — Additional file 1: Appendix 1. Detailed Methods. Appendix 2. Pre-Interview Survey. Appendix 3. Semi-Structured Interview Guide. [file 12889_2021_10449_MOESM1_ESM.zip › additional file(s)/Appendix 2. PreInterview Survey_ESM.docx]

**Appendix 2. Pre-Interview Survey**

**INFORMATION ABOUT AGENCY/ORGANIZATION**

1. Name of agency/organization: _______________________
2. When was the agency/organization established? _______________________
3. How many staff members does your agency have?

[] Less than 5 [] 6-10 [] 11-30 [] 31-60 [] 61-80 [] 81-100 [] More than 100

1. Is your agency/organization on the Supportive Housing Service Master Agreement (SHMSA)?

[] Yes [] No

1. What is your agency’s Target Population(s)? (Check all that apply)

[] Homeless [] Mental Health [] Perinatal [] Substance use

[] Medically complex [] Justice Involved [] Other: ____________________

1. What is your agency’s service area(s)? (Check all that apply)

[] SPA 1 [] SPA 2 [] SPA3 [] SPA 4 [] SPA 5 [] SPA 6 [] SPA 7 [] SPA 8

1. Is your organization receiving Los Angeles County funding to provide direct services?

[] Yes [] No

- 1. If yes, what type of funding is your organization receiving? (Check all that apply)

| [] LAC Department of Health Services  [] LAC Probation Department  [] LAC Department of Public Health  [] LAC Department of Mental Health  [] LAC Department of Children and Family Services  [] LAC Workforce Development, Aging, and Community Services  [] Los Angeles Homeless Services Authority | [] LAC Board of Supervisors Discretionary Funding  [] Federal Grants  [] State of California Grants/Contracts  [] Donations  [] Foundation Grants  [] Insurance Reimbursement for Services  [] Other: __________________________ |
| --- | --- |

1. Are there organizational capacity needs that your program/agency has? [] Yes [] No
   1. If yes, identify up to 10 capacity building needs your program/agency has:

| [] Staff Recruitment and Retention  [] Defining Staff Role, Responsibility, and Onboarding Models  [] Training to Work with Special  Populations  [] Staff Professional and Leadership Development  [] Employee Housing and Social Service Needs | [] Supporting Diversity and Equity in the Organization  [] Change and Growth Management  [] Executive Leadership Coaching  [] Recruitment and Retention of Board Members  [] Quality Improvement | [] Strategic Planning  [] Facility Upgrades and/or  Office Expansion  [] Obtain Appropriate Liability Insurance Coverage  [] Coalition and Collaboration Development |
| --- | --- | --- |
| [] Creating or Updating Financial Management Practices and Policies  [] Fundraising and Sustainability Plans  [] Support for new organizations interested in receiving county contracts  [] Support for organizations providing subcontracts | [] Fiscal Sponsorship and/or Subcontracting Opportunities  [] IT Infrastructure Development  [] Improve Technology and Data Privacy Infrastructure  [] Service Delivery Data Evaluation | [] Knowledge on Data Sharing Relationships  [] Technical Assistance with County Contracting Processes and Compliance  [] Support to grow from a subcontractor to a primary contractor  [] Other |

1. Are you aware of the Medicaid Waiver, Whole Person Care Program (WPC)?

[] Yes [] No

- 1. If yes, when did your agency start enrolling or referring participants into Whole Person Care?____________________

1. What specific WPC program does your agency work with?

| [] Homeless Care Support Services  [] Recuperative Care  [] Benefits Advocacy  [] Intensive Services Recipients (ISR)  [] Community Re-Entry  [] Residential Vocational Education Centers  [] Youth Diversion and Development  [] SUD Engagement, Navigation & Support | [] Tenancy Support Services (TSS)  [] Residential and Bridging Care (RBC)  [] Kin through Peer (KTP)  [] Transitions of Care (TOC)  [] Enhanced Care Coordination- County Jails  [] Innovative Employment Solutions  [] MAMA’s Visits  [] Sobering Center | [] Re-Entry Post Release Adult Community Referral  [] Re-entry Pre-Release Enhanced Enrollment  [] Juvenile Re-entry Aftercare  [] Re-entry Adult Extended Care  [] Re-entry Post Release Adult Jail Referral |
| --- | --- | --- |

1. To what degree is your agency working with other programs, agencies, and/or organizations to enroll clients?

[] Always [] Very Often [] Sometimes [] Seldom

[] My agency does not work with others to enroll clients

1. To what degree is your agency working with other programs, agencies, and/or organizations to deliver services to your clients?

[] Always [] Very Often [] Sometimes [] Seldom

[] My agency does not work with others to deliver services to clients

**INTERVIEWEE DEMOGRAPHICS**

1. Title/Role of interviewee: ____________________________
2. Race/Ethnicity:

[] White [] Hispanic/Latino [] Black/African American

[] Native American/American Indian [] Asian/Pacific Islander

[] Other:_____________

1. Gender: _________________________
2. Highest level of training/Degrees: ____________
3. Years in this field of social/behavioral health services: _______
4. # of years affiliated with the agency: [] <1 year [] 1-5years [] >5 years
5. Do you live in the Service Planning Area (SPA) your agency is serving?

[] Yes [] No

- 1. If yes, how long have you lived there? ___________

1. Do you have a similar lived experience as the population your agency serves?

[] Yes [] No

- 1. If yes, years of lived experience with the population your agency serves: _________
