# Supplementary material for: Community-based organizations’ perspectives on improving health and social service integration
Source: BMC Public Health. 2021 Mar 6;21:452. doi: 10.1186/s12889-021-10449-w (PMC7937223; doi:10.1186/s12889-021-10449-w)
Supplement: Supplementary file 1 — Additional file 1: Appendix 1. Detailed Methods. Appendix 2. Pre-Interview Survey. Appendix 3. Semi-Structured Interview Guide. [file 12889_2021_10449_MOESM1_ESM.zip › additional file(s)/Appendix 3. Semi-Structured Interview Guide_ESM.docx]

**Appendix 3. Semi-Structured Interview Guide**

**Introduction**

Thank you for taking the time to participate. In this conversation, I hope to gain your perspective and learn from your experience as a representative of a community agency in regards to the clients your agency cares for, how your services are delivered, the desired goals for your clients, and the partnerships you may have with other organizations to achieve your goals in the context of the recent implementation of the Whole Person Care Los Angeles Program (WPC). Once we have conducted all of interviews, our team will analyze the data, and host a conference with all community partners to share the results to get your feedback before we report it elsewhere.

I will record our conversation and it should take about 60 minutes. Please feel free to answer whichever questions you are comfortable with and avoid those that you feel uncomfortable with. Do you have any questions or concerns before we proceed?

**QUESTIONS**

**Information about interviewee:**

1. Please tell me about your role within [*the name of the program/agency/organization*]
   1. *Follow up:* What were the main factors that drew you to this work?

**Description of targeted populations needs/assets:** For the next several questions, please consider your experience working with the [*name of the program/agency/organization]* and the [*homeless, mental health, perinatal, substance use, justice involved, or medically complex*] populations that it serves.

1. What do you think are the top 3 characteristic strengths of your clients that affect their lives?
   1. *Follow up:* How do you think [*name strengths listed*] affect their lives?
   2. *Follow up:* How do you think the neighborhood where your clients live influences the [*name strengths listed*]?
2. What do you think are the top 3 needs of these clients that affect their lives?
3. *Follow up:* How do you think [*name needs listed*] affect their lives?
4. *Follow up:* How do you think the neighborhood where your clients live influences these needs?

**Description of program/goals & vision:** For the next several questions, I would like to explore how [*name of the program/agency/organization]* functions and its goals/visions.

1. Based on your experience with [*name of program/agency/organization*], what is the primary role of your [*program/agency/organization]* in helping the [*homeless, mental health, perinatal, substance use, justice involved, or medically complex*] population?
   1. Briefly, walk me through the process that a client undergoes to receive services from your [*program/agency/organizations*].
      1. *Follow up:* How are they enrolled in [*name of program*]?
      2. *Follow up:* What resources are provided to the clients?
      3. *Follow up:* How, if at all, is data about the clients collected? How is it used?
2. What are the top 3 goals for your program in helping the population it serves?
   1. *Follow up*: How is your daily work informed by these goals?
   2. *Follow up:* What are the biggest challenges to reaching these goals? Why?
      1. *Follow up:* How would you fix these challenges to achieve the program goals?
   3. *Follow up:* What have been the most useful things that help the program in reaching its goals?
      1. *Follow up:* How do you think [*list facilitators mentioned*] can be better used to address the goals of your program?
   4. *Follow up:* Considering your responses to the [*name strengths and needs listed prior*] of the clients your [*program/agency/organization*] serves, how do you think they affect its goals?

**Partnerships- other agencies/health systems/WPC:**

1. Let’s shift gears and discuss partnership with other organizations. How, if at all, do you work with other organizations or agencies?
2. *Clarification/Follow up:* Organizations or agencies such as CBOs, private foundations, health care systems, Los Angeles County DHS, DMH, DPH.
3. *Follow up:* In what ways does your work with these other organizations affect the goals of [*name of program/agency/organization*]?
4. Based on the survey, you said you are aware of the Whole Person Care Program and are affiliated with [*name of WPC program*]. Can you tell me how [*name of program/agency/organization*] works with WPC?
   1. In what way, if at all, has WPC changed [name of program/agency/organization’s] ability to achieve the goals that you mentioned before (*list the goals mentioned*)?
      1. *Follow up:* Thinking about how you described the processes clients go through to receive services from [*name of program/agency/organization*], how has WPC changed:
         - - the number of individuals enrolled into your program?
           - how individuals enroll in your program?
           - the ease or difficulty of enrolling patients in your programs?
           - your agency’s capacity/resources to address the needs of [homeless persons, individuals with mental illness, or pregnant women]?
           - the number and type of collaborations between your agency and other organizations in Los Angeles County?
5. What are the 3 biggest challenges that WPC creates for [*name of organization*] to help the [*homeless, mental health, perinatal, substance use, justice involved, or medically complex]* population that it serves?
6. *Follow up:* How do you think these challenges could be overcome?
7. What are the 3 main things that WPC does that helps [name of organization] to serve the [homeless, mental health, perinatal] population that it serves?
8. *Follow up:* How do you think these [*name things mentioned*] could be further built upon?
9. Considering the challenges and strengths that WPC creates for your organization and others, what needs to be done to continue to support the positive changes that have occurred?
   1. *Follow up:* How should partnerships evolve to ensure that [*name of organization*] could continue to provide services to its clients?

**Conclusion:**

1. If you were asked to tell leaders of Whole Person Care about [*name of program/agency/organization*] and the clients you serve what would you tell them?
2. Is there anything else about [*name of program*], the clients you serve, and/or WPC that you have not discussed that you want to share?

What you shared is very valuable and again, thank you very much for your time. I am turning off the recorders now.
